# Reading spike timing without a clock: intrinsic decoding of spike trains

**DOI:** 10.1098/rstb.2012.0467

**Published:** 2014-03-05

**Authors:** Stefano Panzeri, Robin A. A. Ince, Mathew E. Diamond, Christoph Kayser

**Affiliations:** 1 Institute of Neuroscience and Psychology, University of Glasgow, Glasgow G12 8QB, UK; 2 Center for Neuroscience and Cognitive Systems @UniTn, Istituto Italiano di Tecnologia, Via Bettini 31, 38068 Rovereto, Trentino, Italy; 3 Tactile Perception and Learning Laboratory, International School for Advanced Studies, 34136 Trieste, Italy; 4 Bernstein Centre for Computational Neuroscience, Tübingen, Germany

**Keywords:** information theory, sensation, neural code, decoding, spike patterns, oscillations

## Abstract

The precise timing of action potentials of sensory neurons relative to the time of stimulus presentation carries substantial sensory information that is lost or degraded when these responses are summed over longer time windows. However, it is unclear whether and how downstream networks can access information in precise time-varying neural responses. Here, we review approaches to test the hypothesis that the activity of neural populations provides the temporal reference frames needed to decode temporal spike patterns. These approaches are based on comparing the single-trial stimulus discriminability obtained from neural codes defined with respect to network-intrinsic reference frames to the discriminability obtained from codes defined relative to the experimenter's computer clock. Application of this formalism to auditory, visual and somatosensory data shows that information carried by millisecond-scale spike times can be decoded robustly even with little or no independent external knowledge of stimulus time. In cortex, key components of such intrinsic temporal reference frames include dedicated neural populations that signal stimulus onset with reliable and precise latencies, and low-frequency oscillations that can serve as reference for partitioning extended neuronal responses into informative spike patterns.

## 1. Introduction

Brain functions such as perception and action are based on neural representations of the external world. An important question is therefore how the characteristics of external events, for example sensory stimuli, are represented by patterns of neural activity in the brain.

Neural responses can vary over short time scales—under 10 ms—and substantial evidence suggests that the temporal structure of neural activity encodes sensory information. In particular, knowledge of the precise spike times with respect to the time of stimulus presentation adds information about sensory stimuli that is irremediably lost if spike times are sampled with insufficient temporal resolution [1,2]. Evidence that precise spike times carry sensory information above and beyond the information contained in spike counts computed over longer windows has been reported across different brain structures, from peripheral to cortical areas and across sensory modalities [2–13].

The structure and information content of time-varying spike trains are typically analysed by aligning spikes and sensory events using a laboratory-based computer clock that registers stimuli and neural events with supreme accuracy. However, accessing information contained in the temporally precise relationships between the timing of stimuli and that of the elicited spikes demands that this temporal precision is preserved in the operations made to read out these responses. This requires that the decoder perform at least two computations. First, the decoder must obtain precise knowledge about the timing of sensory events (e.g. stimulus onset or a reference point during the stimulus time course). Second, the decoder must have access to a representation of time intervals with some degree of precision. These two basic operations are used for virtually any analysis of spike patterns. They are used, for example, when computing a classical peri-event time-histogram using a division of time into smaller and equally spaced time bins that are aligned to stimulus onset.

These considerations raise the important question of how the brain may succeed in interpreting the information carried by the temporal variations of neural responses without the benefit of a computer clock measuring perfect time intervals and providing the exact time of stimulus presentation [14–16]. In conditions when the motor system actively initiates or modulates the external event [15,17,18], sensory systems may possibly receive a motor efference copy that reduces temporal uncertainty about stimulus timing [19–21]. However, when sampling is not actively initiated and when the stimulus appears suddenly and unpredictably, such efference mechanisms are not available and the system requires an intrinsic temporal reference.

How the brain maintains a representation of time intervals also remains unclear [22]. Some suggest the existence of a common specialized—and perhaps centralized—mechanism for all or most timing operations [23]. This view is difficult to reconcile with the variety of time scales at which spike times carry information. For example, in cortex, the temporal precision by which spike trains carry information varies with the sensory modality: somatosensory stimuli are encoded with millisecond-scale precision [6], auditory stimuli encoded with precision of 5–10 ms [24,25], and most visual studies report cortical encoding with precision of the order of several tens of milliseconds [2]. Furthermore, even within a sensory modality, the dominant time scale varies with the dynamics inherent to the stimulus, with more rapidly developing stimuli requiring a finer temporal resolution for decoding [24,26]. Given this heterogeneity of time scales, it is likely that the ability to measure timing and to represent stimulus time is distributed among different structures [27,28]. In particular, it is possible that the reference frames needed to decode spike patterns are provided by the activity of the local network itself [14,29–32]. Thus, a hypothesis that we explore here is that sensory networks interpret time-varying responses using an internally available reference frame.

In this review, we focus on the problem of how decoders may extract information from spike times using different reference frames. We first describe relevant analytical approaches to address this problem and we then review recent studies investigating intrinsic reference frames derived from local network activity.

## 2. Information theoretic metrics to evaluate different codes and reference frames

To study internal reference frames for sensory decoding, it is necessary to have quantitative tools to assess the amount of information carried by different putative coding schemes. Shannon information, abbreviated hereafter as information, offers a rigorous measure to compute single-trial stimulus discriminability2.1
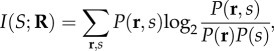
where *P*(**r**,*s*) is the joint probability of presenting a stimulus *s* and observing a response **r**, and *P*(**r**), *P*(*s*) are the respective marginal probabilities. Information quantifies the reduction of uncertainty (i.e. the gain in knowledge) about the stimuli obtained from a single-trial observation of a neuronal response (averaged over stimuli and responses). Information is measured in bits (1 bit corresponds to a reduction of uncertainty by a factor of two) and is an upper bound on the amount of knowledge about stimuli that can be extracted by any decoding algorithm operating on neural responses. The fact that mutual information quantifies single-trial stimulus knowledge is particularly appealing because neural systems usually must discriminate or identify stimuli on a single encounter.

By evaluating the information carried by neural codes **r** based on different response aspects (timing or number of spikes) and defined relative to different reference frames, one can evaluate the capacity of different candidate neural codes. Below, we specifically compare the information obtained from responses **r** quantified using the experimenter's clock with responses **r** defined using an internal reference signals.

Owing to the difficulties of computing stimulus–response probabilities from finite amounts of experimental data [33], information metrics are sometimes computed using an intermediate decoding step. In this approach, one first computes for any given response **r** the most likely stimulus *s^P^* that elicited this response using a decoding procedure (e.g. template matching) and cross-validation [34,35]. Then, the information extracted through the stimulus reconstruction scheme can be quantified as follows [34]:2.2
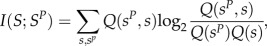
where 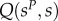 is the joint probability that in a trial the decoding procedure reports the presence of stimulus 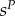 and the true presented stimulus is *s*. The decoded information 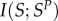 quantifies (in bits) the average knowledge gained, per trial, when predicting the stimulus using a specific algorithm, and takes into account both the fraction of correct decoding and the spread of the decoding errors.

## 3. Decoding first post-stimulus spikes in somatosensory cortex without knowledge of stimulus time

We begin by considering how neurons in the whisker ‘barrel’ field of primary somatosensory cortex encode the identity of a deflected whisker. Knowing which whisker has contacted an object is thought to be part of the process by which rats localize objects, and hence a key variable encoded in this area [18,36–38].

**Figure 1.**
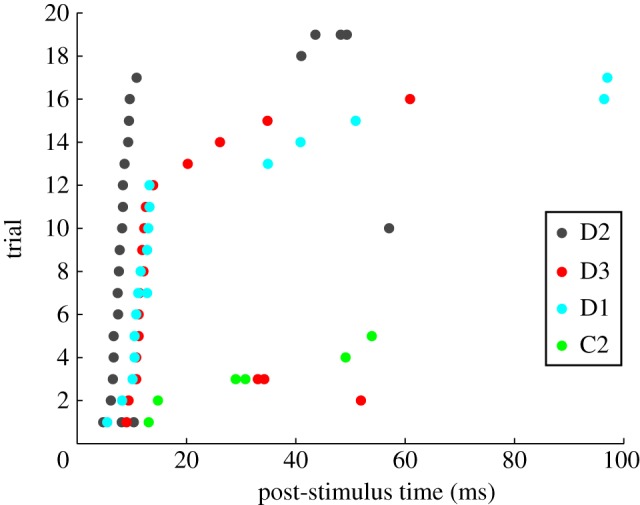
Latency coding of stimulus location in rat somatosensory cortex. Raster plot of the spike times of one example neuron recorded from barrel-column D2 in rat somatosensory cortex in response to a sudden whisker deflection applied at time 0. Each dot represents the time of a spike, and each row represents a different stimulus presentation (trials ordered by increasing first-spike latency). Responses to deflection of different whiskers are plotted using different colours. *X*-axis represents post-stimulus time. Vibrissae C1-3, D1-3, E1-3 were stimulated in this dataset, but only trials in response to deflection of the four whiskers that elicited a significant response are shown. Figure prepared from data published in [6].

In previous work, we analysed the responses, recorded in anaesthetized animals from barrel cortex neurons, to discrete single-whisker deflections. We found that spike times measured with resolution of 5 ms or finer allowed the extraction of approximately 50% more information about the identity of the stimulated whisker than the information obtained when counting spikes over post-stimulus windows of a few tens of milliseconds [6,39]. Moreover, almost all the information about whisker identity provided by the whole train of spikes emitted after stimulus presentation was carried already by the timing (or latency) of the first post-stimulus spike. In other words, the timing of the first post-stimulus spike carried all the information about stimulus identity, whereas all successive spikes carried ‘old news’. This is owing to the extremely precise whisker-dependent first-spike latency of these neurons (see example in figure 1). Given that the information-bearing variable (first-spike latency) is not even defined without knowledge about stimulus timing, this system proves particularly interesting for understanding how information in spike times may be decoded using an internal reference frame.

**Figure 2.**
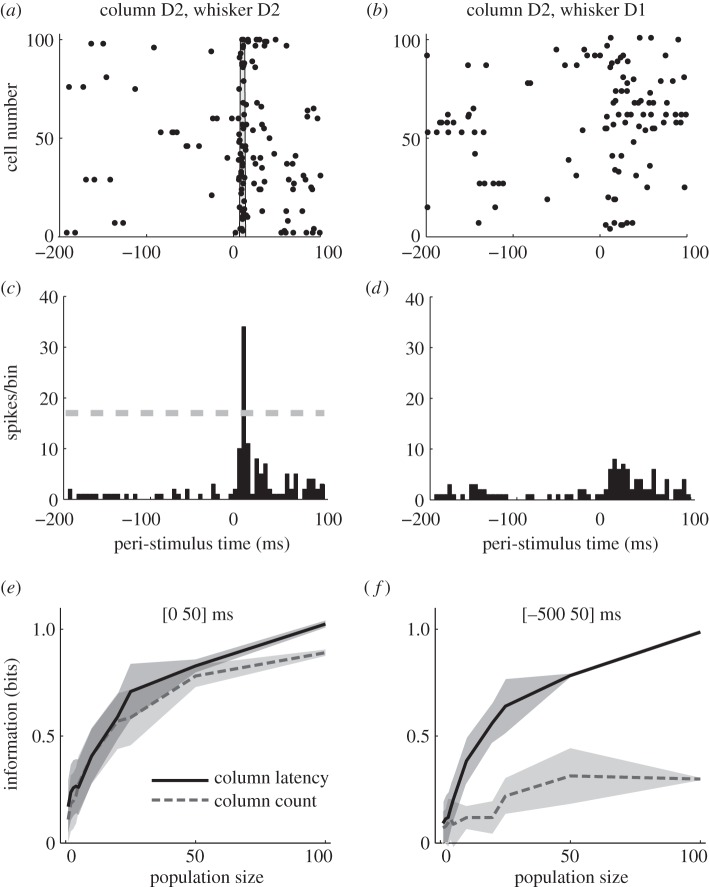
Extracting clock-free information about the identity of a stimulated whisker from single-trial responses of a barrel column to whisker deflection (*a*) Raster plots of the spike times emitted in a single example trial by 100 neurons recorded non-simultaneously in barrel-column D2 around the time of deflection of the principal whisker D2. The grey area denotes the time window when many neurons synchronously fire their first post-stimulus spike (*b*) Raster plots of the spike times emitted in a single example trial by 100 neurons recorded non-simultaneously in barrel-column D2 around the time of deflection of the whisker D1 (*c*) The time course (sampled in 5 ms bins) of the summed activity of the whole population of D2 neurons around the time of deflection of D2 whisker in the same trial plotted in (*a*). The dashed horizontal line plots the threshold used to detect a ‘CSR’ event. (*d*) The time course of the summed activity of the whole population of D2 neurons around the time of deflection of D1 whisker in the same trial plotted in (*b*). (*e,f*) The information about whether whisker D1 or D2 was deflected, obtained by the summed counts (dashed line) or the latency of synchronous population activity (solid line) of a population of neurons located in a single column (barrel D2). Panels (*e*) and (*f*) report, respectively, information values which were obtained when using either the [0 50] or the [−500 50] ms peri-stimulus time range. Results are plotted as average (±s.d.; shaded area) across all analysed subgroups of cells with the specified population size. Figure redrawn from [40].

The key may be in the *relative* timing of spikes. To illustrate this, let us first examine the single-trial response of the entire population. Figure 2*a* shows the responses of 100 non-simultaneously recorded neurons in column D2 to one deflection of its topographically matching whisker, D2, at time *t* = 0. Approximately 10 ms after stimulus onset many neurons fired nearly simultaneously (grey area in figure 2*a*). By 50 ms after stimulus onset, activity had returned to baseline (figure 2*a*). Responses of these neurons to deflection of a different, non-topographically matching whisker (D1) are shown in figure 2*b*, where spikes were fewer and more distributed in time. We have previously shown that summing the population activity of neurons located within the same column does not lead to a loss of information about whisker identity [41]. In the following, we therefore consider the summed population activity within a column, which is shown in figure 2*c,d*. This summed population activity shows a stronger, shorter latency response to the topographically matching whisker.

It is tempting to assume that coarse codes, for example spike count over relatively large time windows, may be more robust to temporal uncertainty about stimulus timing than codes relying on precise spike timing. To evaluate this hypothesis, we investigated how decoding accuracy using the summed spike count over long windows of the columnar population depends on knowledge of stimulus timing. A decoder defining the response **r** (equation (2.1)) as a columnar population spike count exploits the fact that in the few tens of milliseconds following stimulus onset more spikes are emitted in response to stimulation of the topographically matching whisker than to any other [6,42]. This suggests that spike counts registered in some post-stimulus window should discriminate the stimulated whisker well. For the information analysis, we considered a population of *n* sequentially recorded neurons in column D2 and varied *n* parametrically from 1 to 100. We then computed how much information the columnar spike count encoded about the stimulated whisker (D1 or D2), with perfect discrimination corresponding to 1 bit of information.

We compared two cases. In the first case, we assumed that the observer has precise knowledge about the time of whisker deflection. Therefore, it counted the spikes in the ‘correct’ post-stimulus window ([0 +50] ms) in which all post-stimulus spikes are emitted. In this case, the resulting population spike count carried a relatively high amount (0.8 bits) of stimulus information. In the second case, we assumed that the decoder has imprecise knowledge about whisker deflection time and, as a consequence, observes both the period of spontaneous activity preceding the stimulus as well as the stimulus-evoked response. This observer hence quantified the columnar population responses in the time window [–500 + 50] ms around stimulus time. As spike counts are modulated by stimulus identity for only a short window after whisker deflection, and rates of spontaneous spikes vary randomly across trials and stimuli, the stimulus discriminability by this spike count became much poorer (0.3 bits). The conclusion, perhaps counterintuitive, is that integrating columnar spike counts over long windows is not an effective way to make the neural code robust to errors in the estimation of the stimulus time. In this neural system, population spike counts can only be decoded provided there is sufficiently precise knowledge of the stimulus time to tell when to count spikes.

An alternative hypothesis is that registering the population response with high temporal precision can actually make the code more robust to errors in estimating the stimulus timing. Figures 1 and 2*a,b* show that the first spikes emitted by the 100 neurons in barrel D2 after stimulation of their topographically matching whisker are much more precisely timed than second spikes or spikes emitted in response to a non-topographically matching whisker. This suggests that one can use the precisely aligned response latencies of individual neurons to define a ‘columnar synchronous response’ (CSR) event characterized by the firing of at least a certain fraction *f* (here 17%) of neurons within a short window Δ*t* (here 5 ms) (figure 2*c*). We searched for CSR events by moving a sliding window of size Δ*t* throughout the peri-stimulus time. The event occurred only in the window [+10 +15] ms after deflection of the topographically matching whisker and never during spontaneous activity or after stimulation of a non-topographically matching whisker [40]. This result is exemplified in one selected trial in figure 2*c,d*, but it held perfectly for all trials. To compute the information encoded by the columnar latency, we called the time at which the first CSR event was detected in a given trial the ‘columnar latency’ and we used this as neural code **r** in equation (2.1). We then computed information from the columnar latency and found that it surpassed that of spike counts and reached the 1 bit value corresponding to perfect discrimination (figure 2*e*). Moreover, and unlike the spike counts, if enough cells were included in the calculation of CSR events then the information carried by the columnar latency was robust to the insertion of long periods of spontaneous activity in the window to be analysed (figure 2*f*).

Columnar latency reflects two aspects: CSR presence and CSR timing. An interesting question regards the role of these two aspects in the information advantage of the columnar latency over the columnar spike count. For small number of neurons, CSR events are noisy, so CSR timing can add some information about whisker identity to that carried by CSR presence. This expectation is compatible with the finding that for single neurons or pairs the first-spike latency carries approximately 50% more information about whisker identity than counting the spikes in the early response part [6,39,40]. When using 100 neurons per column, the detection of CSR is 100% robust, and so CSR timing cannot add much information about whisker identity. However, with 100 neurons per column the timing of CSR events is reproducible in each trial with millisecond precision [40], showing that CSR timing faithfully signals the timing of deflection of the topographically matched whisker.

These considerations suggest that in this dataset for large enough numbers of neurons the stimulus-dependent millisecond-precise latency aligns the spikes of different neurons in the same column to fulfil two information-coding goals: the presence of a simultaneous co-activation in a given column signals the stimulation of the topographically matching whisker (and thus codes whisker identity), whereas the timing of this co-activation signals the timing of this stimulation (and thus codes stimulus time).

These calculations do not take into account the effects of correlated noise, because they are based on a pseudo-simultaneous response array. To test for the effect of correlated variability, we generated simulated correlated spike trains that matched exactly the true population-averaged time-dependent firing rate of the neurons (sampled with 1-ms bins) and the true pairwise cross-correlations of neuronal pairs within the same column in each bin [39,40]. When the simulated population of 100 cells per column with realistic correlation values was tested, performance of the columnar latency difference decoder was unchanged [40], suggesting that the results presented here would only be mildly affected by correlated noise.

Here, we presented results on the robustness of the codes to ‘backwards’ errors in knowledge of stimulus time that led to including in the analysis periods of prestimulus activity. This was because we were interested in the robustness of first-spike detection to spontaneous activity. However, it is important to bear in mind that ‘forward’ errors in the stimulus time (i.e. when stimulus time is estimated to happen later than it really does) can also be profoundly detrimental for reading out the information. For example, for transient responses as those of the barrel cortex neurons analysed here (figures 1 and 2), clearly a forward error of as little as 20 ms in detecting the correct stimulus start time would mean a loss of essentially all information for both spike times and spike counts.

Interestingly, similar mechanisms based upon short-time integration of pooled population activity have been found across different stages of the whisker pathway in a number of behavioural conditions [43–45]. This suggests that a similar decoding mechanism may apply at several stages of the whisker-processing pathway. In conclusion, the above analysis suggests that detecting the millisecond-precise latency of population activation leads to a reliable and highly informative decoding of stimuli that can be more robust than spike counts to uncertainty about stimulus time.

## 4. Stereotyped neurons as population reference for stimulus onset in auditory cortex

**Figure 3.**
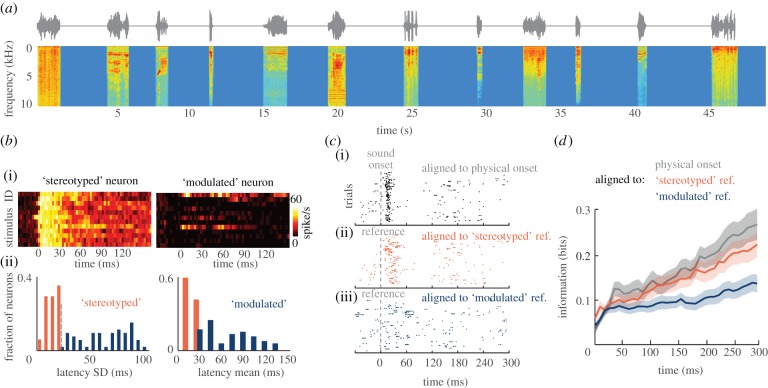
Stereotyped neurons as population reference for stimulus onset in auditory cortex. (*a*) Sound wave and spectrogram for the auditory stimulus sequence on one example trial. Twelve different natural sounds (stimuli) were presented in pseudo-random sequence and with random intersound intervals (blue periods) on each trial. The sound wave is shown above the spectral representation (red colours indicate high power). (*b*) (i) Time course of trial-averaged responses to all 12 stimuli (each row represents a different stimulus) for one stereotyped (left) and one modulated (right) neuron. Time *t* = 0 corresponds to sound onset. (ii) Distribution of the latency variability (standard deviation across trials) and mean latency across neurons. Stereotyped and modulated neurons are colour-coded in red and blue, respectively, the dashed line in the left histogram indicates the threshold used to separate (i.e*.* define) the two groups. (*c*) Example response from one modulated neuron after being aligned to different reference frames. (i) Spike raster when aligned to stimulus onset time (*t* = 0). (ii) The same response but with each trial aligned to the response onset of a simultaneously recorded stereotyped reference neuron (here *t* = 0 corresponds to the onset latency of the reference neuron). (iii) The same response aligned to the onset of a modulated neuron. While the stereotyped reference preserves the temporal shape of the stimulus locked response, the use of a modulated neuron as reference results in a much more dispersed spike raster. (*d*) Information about stimulus identity obtained using the three considered reference frames in progressively longer time windows (starting at *t* = 0 and ending at each indicated time point). Lines denote the mean and shaded areas the standard error (s.e.m.) across the population (*n* = 48) of modulated neurons. Information was computed with a linear decoder and equation (2.2). Figure redrawn from [46].

The above suggests that a neural population event could provide an estimate of the time of the stimulus, which could then be used to measure the relative timing of subsequent spikes. To understand how such a relative coding scheme could be implemented as a general principle of information coding in cortex, it is helpful to understand whether and how explicit stimulus timing signals could be used in other cortical sensory systems. To be a plausible ‘clock’, that signal must be sufficiently robust to allow the extraction of information also about complex natural stimulus features, in the alert animal and without any external predictive clues about stimulus timing. We investigated the viability of a relative coding scheme and its robustness with regard to these requirements in the auditory cortex of awake primates [46]. To this end, we recorded the responses of single neurons from primary auditory cortex to naturalistic sounds (conspecific vocalizations, vocalizations or noises of other animals) using a paradigm minimizing predictive cues about stimulus onset (figure 3*a*).

We first characterized the response latencies of these neurons. Specifically, we detected the single-trial response latency using a statistical algorithm and then computed the trial-to-trial variability of the latency (computed as the standard deviation of the latency across all trials to each stimulus, and then averaged across stimuli). This revealed a clear dichotomy within the population: some neurons achieved very low latency variability, while most others attained much higher levels of variability. For analysis, we partitioned the population into two subpopulations using a single criterion: a threshold applied to the latency variability (figure 3*b*). The subpopulation of neurons with low latency variability was termed ‘stereotyped’ neurons to reflect the fact that they all had similar onset latencies across stimuli. These made up 24% of the total population. The neurons classified as those with larger latency variability were termed ‘modulated’ neurons. This grouping revealed further key differences: stereotyped neurons responded to all tested sounds, whereas modulated neurons responded only to some sounds, and stereotyped neurons responded with very short latencies. The mean latencies of stereotyped neurons (21.7 ± 0.8 ms) were much shorter than those of modulated neurons (72.0 ± 4.6 ms; two-sample *t*-test *p* < 10^−7^). Example responses of one stereotyped and one modulated neuron are shown in figure 3*b*.

The stereotyped neurons stand out because of their rapid, reliable and invariant early responses. Their fast and non-specific responses make them natural candidates as an intrinsic time reference frame, relative to which the time-varying responses of other stimulus-modulated neurons could be measured. We tested this hypothesis by constructing two candidate codes **r** based on the spike times of modulated neurons using two reference frames: the precise stimulus onset time (external reference) or the latency of a simultaneously recorded stereotyped neuron (internal reference). We found that the responses of modulated neurons exhibited temporally precise stimulus-modulated patterns of spike times relative to the stimulus onset (figure 3*c*), emphasizing the high information content of precise spike timing in auditory cortex [24,47]. These temporal response patterns were partly preserved when the responses of modulated neurons were aligned to the single-trial onsets derived from a simultaneously recorded stereotyped neuron (figure 3*c*). Owing to the temporal reliability of stereotyped neurons, little of the information about sound identity carried by spike times relative to stimulus onset was lost (figure 3*d*). Importantly, using another simultaneously recorded modulated neuron as an intrinsic temporal reference proved much worse (figure 3*d*). This shows that the selective pooling of stereotyped neurons is necessary to form a reliable internal indicator of stimulus timing. Using a modelling approach, we found that pooling about 25 stereotyped neurons was sufficient to produce a reference signal that allows extracting more than 95% of the full information provided by spike times measured relative to the precise stimulus onset [46]. The relative timing of neural responses to an intrinsically defined population event can hence constitute a highly informative code also in the alert animal and for complex and suddenly appearing stimuli.

## 5. Network oscillations as intrinsic reference frame for partitioning spike sequences in auditory cortex

The problem of temporal reference frames not only includes the intrinsic detection of stimulus appearance, but also the internal representation of time intervals needed to partition extended neural responses into informative spike patterns during prolonged stimuli. A relevant example of such continuous and lasting stimulation comes from the auditory system, which in real-life conditions is often faced with a stream of sounds and has to represent individual sound objects within a continuously evolving environment [48,49]. Examples are individual words in a spoken sentence, a melody in a song or individual sounds appearing in a cacophony of environmental noises. We performed a separate study to test whether signals derived from the cortical network can serve to define an intrinsic time frame relative to which longer sequences of spike times can be interpreted.

**Figure 4.**
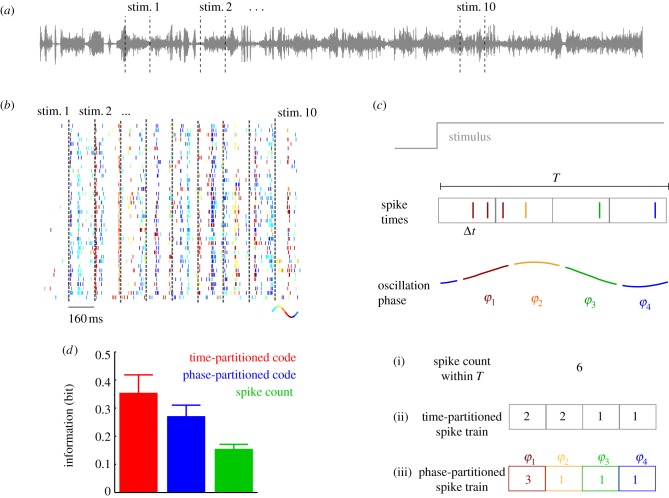
Network oscillations as an intrinsic reference frame for partitioning spike sequences in auditory cortex. (*a*) Sound wave of the 52 s sequence comprising natural and environmental sounds presented during the experiment. Dashed lines illustrate the random selection of 10 stimulus epochs used for the decoding analysis (stimulus epoch duration not to scale). (*b*) Spike raster from one example neuron with spikes colour-coded according to the phase of the concurrently recorded 2–6 Hz LFP during multiple repetitions of each stimulus epoch. (*c*) Schematic of the different partitioning schemes within a stimulus epoch of width *T*. The timing of spikes can be measured by temporal binning relative to stimulus onset. This ‘time-partitioned code’ is defined as the vector consisting of the number of spikes per time bin. Alternatively, the timing can be measured relative to an intrinsic slow oscillatory signal. Here, the phase of such an oscillation was divided into four phase quadrants and spikes are colour-coded by their respective phase angle. This ‘phase-partitioned’ code is defined as the vector consisting of the number of spikes per phase range. For comparison, a ‘spike count’ code was defined as the total number of spikes within the window *T*. (*d*) Stimulus information provided by each code across neurons sampled in auditory cortex (mean ± s.e.m.; *n* = 40). Figure is redrawn from [50].

To this end, we studied the responses of neurons recorded from monkey primary auditory cortex during the presentation of a 52 s continuous sequences of naturalistic sounds, such as animal calls and environmental sounds, whose sound waves are illustrated in figure 4*a* [25,50].

Figure 4*b* displays the response of one example neuron to different chunks of the auditory stimulus (each chunk represents a different stimulus epoch for computation of information and is indicated as ‘stim. 1’, ‘stim. 2’, etc. in figure 4*a,b*). The raster plots display the spike trains evoked on individual repeats of the sound sequence. Previous work showed that partitioning these responses using the laboratory clock to construct perfectly spaced and stimulus-aligned time bins (*time-partitioned* spike trains; figure 4*c*) reveals spike patterns that carry information with high temporal precision (here few milliseconds, e.g. [24] and figure 4*b*).

We then investigated how one could obtain approximate representations of such time-partitioned responses using a reference frame defined purely in terms of intrinsic network activity. In the first attempt, we considered whether neural responses allow the identification of specific points during the long stimulus sequence, for example by synchronous population activity (as we exemplified above for the case of barrel cortex). However, the responses of different neurons during this long stimulus sequence proved quite heterogeneous, and moments of high firing rate for defining a population signal were not shared by a sufficiently large fraction of neurons. Similar heterogeneity has also been observed in the whisker pathway when using stimuli with complex dynamics [45,51], suggesting that synchronous population events (such as the CSR above) are more robust for isolated stimuli than during prolonged stimulation with complex dynamics. As an alternative, we then considered slow oscillatory network activity, which has previously been suggested as potential reference signal for neural processing [52]. Specifically, we focused on rhythms with cycle lengths of 100 ms or longer, such as delta or theta bands, which are often observed in sensory cortices during naturalistic stimulation [25,53,54].

We hence asked whether the timing of network oscillations, defined by their phase, allows the partitioning of longer sequences of spikes. Slow rhythmic network activity in the auditory cortex entrains to the presentation of natural sounds [25,48,52,53,55]. This implies that the phase of such oscillations can indicate salient points along the stimulus trajectory and becomes reliably associated with the time progression of the continuously varying stimulus [56]. As a result, phase differences can be used to measure time intervals during stimulation. A temporal ordering of spike times within an oscillation cycle can be thus achieved simply by using phase intervals that mimic laboratory-based time intervals. Importantly, such an oscillatory reference frame is intrinsic to the cortical network. Its specific timing parameter, namely the oscillatory phase, is likely to be directly accessible to the local network [57,58]. This is because low-frequency local field potentials (LFPs) reflect changes in neuronal excitability that are spatially coherent over several millimetres [59] and often accompanied by coherent fluctuations of neural membrane potentials [60] whose low-frequency phase provides an effective reference signal for decoding spike information [61]. Given that the majority of synapses are made within local networks [62], pre- and postsynaptic neurons likely have access to the same slow rhythm for the majority of cortical connections.

We used the theta band (2–6 Hz) of LFP as an oscillatory reference to construct time-dependent responses that preserve the sequential order of spikes within the oscillation cycle. In other words, the phase (i.e. the position within a cycle) of the rhythm was used as a temporal reference (i.e. as a virtual ‘time axis’) for the temporal grouping of spikes. It should be noted that given the natural variability of the network rhythm these epochs are not necessarily equally spaced. In the example figure, we used colours to represent four phase quadrants of the oscillation cycle, and we then coloured spikes with the colour of the phase quadrants at which they were emitted (figure 4*b,c*). The phase-partitioned spike train code (abbreviated as *phase-partitioned*) was constructed, within each time window *T*, as the number of spikes occurring within each phase quadrant (figure 4*c*). The temporal organization of the time-partitioned and the phase-partitioned responses is illustrated for an example neuron in figure 4*b*. The stimulus dependence of the phase-partitioned responses is apparent in these coloured raster plots. Stimulus information was calculated using different epochs of the long sound sequence as stimuli (indicated as ‘stim. 1’, ‘stim. 2’, etc. in figure 4*a,b*). We assessed the efficiency of a phase-partitioned code by comparing its information to that of the time-partitioned code and to that of the code based on spike counts within the stimulus window (the latter ignores the time structure of spikes altogether).

The gain of information in the phase-partitioned code over the spike count was large (40%; population mean; figure 4*d*). Moreover, the phase-partitioned code recovered mostly (86%; figure 4*d*) of the information carried by the time-partitioned code. Importantly, we found that the excess information in either partitioning scheme over the spike count was highly correlated across neurons (Spearman's rank correlation *r* = 0.87). Thus, good stimulus discrimination afforded by one partitioning scheme implies good discrimination performance in the other. Noteworthy, for some neurons the information recovered by the phase-partitioned code was higher than that in the time-partitioned code, suggesting that the phase of firing of these neurons relative to the network rhythm was more reliable and stimulus specific than the precise timing to the stimulus itself [50].

These results hence foster the notion that network oscillations may serve as a highly effective, biologically plausible and purely internal reference frame that can create informative spike patterns without requiring an external timing signal.

## 6. Intrinsic reference frames in visual cortex

It is important to consider whether the intrinsic reference frames proposed above generalize across sensory modalities. The existence of internal reference frames has previously been investigated in visual cortex. Shriki *et al*. [63] studied the encoding of visual orientation and also reported a subpopulation of stereotyped neurons with reliable non-stimulus-selective response latency. Similar to our results in the auditory system, these visual ‘stereotyped’ neurons could be used to compute informative spike times from other neurons with longer and stimulus-selective latencies [63]. In addition, we could show that slow visual cortical network oscillations also form a reliable network-intrinsic reference for partitioning extended neuronal responses into spike patterns that carry considerable information about natural video clips [50].

## 7. Discussion

Previous work has accumulated considerable evidence showing that the millisecond-precise timing of spikes may add important information to that already carried by spike counts [1]. However, the existence of information encoded by temporal spike patterns does not guarantee that the nervous system can make use of such temporally precise codes. One commonly raised criticism is that spike-timing information cannot possibly be accessed by a downstream neuron if it is encoded in variables, for example post-stimulus latency, which are defined with respect to external reference frames and as such they are not directly accessible to biological neural networks. As a result, understanding how spike sequences may be decoded purely from intrinsic time reference frames is a key step in linking temporally precise neural codes with behaviour. Studies over the last few years began to investigate this problem, and the results reviewed here provide a series of useful insights.

The first and the most surprising insight is that, differently from what many investigators may believe, decoding by spike count rather than spike timing does not make the readout of the information more robust to imprecisions in the knowledge about stimulus timing. We found that in somatosensory cortex a spike count code could prove more fragile to the uncertainty about stimulus timing than a spike-timing code, mostly because of the time-dependent transient nature of neural responses and the presence of spontaneous activity [40].

The second insight is that the population activity of the network itself can constitute an adequate reference frame for reconstructing informative spike patterns. Considerable information can be recovered under challenging conditions, including natural stimuli with unpredictable onset time [46] or long stretches of natural stimuli [50]. In some cases, internally referenced codes may outperform externally referenced ones [14,30,50]. This may, e.g*.* happen when variations in spike timing are coordinated across neurons owing to a common covarying factor. In this case, spike timing relative to the stimulus is more adversely affected than the relative timing between neurons [64].

The third insight is that the encoding of stimulus time and of stimulus identity seems to some extent to be separated into distinct populations within a single area [46,63]. While some neurons show time-dependent stimulus-selective responses, other neurons with short-latency but stimulus-unselective responses encoded stimulus time [46]. This draws renewed attention to apparently unselective neurons that may have been systematically ignored in previous work. These neurons could act as ‘saliency detector’ neurons and may have the function to ensure that the early post-stimulus part of neural responses (which is the most informative one in many cases [1,6,10,29]) is not missed out. Future work is required to elucidate the exact morphological and biophysical properties and the location within cortical microcircuits of such putative salience detection neurons. Future work also needs to elucidate how these neurons interact with slow network rhythms to collectively form reliable and precise intrinsic temporal reference frames for neural coding.

It is important to bear in mind that the fact that the timing of spikes relative to stimulus timing carries information and that the brain has an effective intrinsic clock to read out this information do not necessarily imply that the brain actually extracts information this way. These two conditions are necessary, rather than sufficient, for spike timing of neurons post-stimulus to be used in brain function. The brain may well extract the same information from other features of population activity. For example, information may be transmitted by stereotyped spatio-temporal patterns of population responses used to tag different stimuli or behavioural conditions and not necessarily emitted with a precise relationship with the stimulus presentation time [65–69].

Further work is required to directly link different candidate neural codes to behaviour. A causal approach to investigate which neural codes are used for behaviour is to manipulate the temporal structure of neural activity and examine how these manipulations cause changes in behaviour. In a series of such studies, rats were trained to discriminate activity patterns induced by electrical microstimulation in sensory cortex [70,71]. These studies demonstrated that small time lags between neural activations, of the order of few to few tens of milliseconds, and similar in principle to the ones reviewed here, can indeed be read out by downstream neurons. A statistical approach to investigate which codes are used for behaviour involves measuring how the information content of different neural codes correlates on a single-trial basis with behavioural performance during perceptual discrimination tasks. This approach would benefit from the ability to record a large number of neurons participating in the task. For cortical systems, this requires further advances in multi-neuron recordings enabling the sampling of sufficiently many neurons with high temporal precision, together with advances in the development of analytical procedures able to extract low-dimensional descriptions of the spatio-temporal pattern of population activity and to relate them to behaviour on a trial-to-trial basis.

The work reviewed here does not tell us about how the computations needed for decoding spike-timing information may be implemented at the biophysical level. Insights about this can be gained by computational models [72]. Sensitivity to temporal spike patterns at different scales, for example, can arise from synaptic mechanisms like short-term depression or facilitation [73,74]. Recent work has emphasized how downstream learning and decoding of temporal patterns of spikes can be implemented by spike-timing-dependent plasticity (STDP). Downstream neural networks endowed with STDP can easily localize a repeating spatio-temporal spike pattern embedded in equally dense background spike trains, even in the absence of an explicit time frame [75]. Such plasticity of decoding mechanisms may be further facilitated by the fact that internally referenced patterns of neural activity, though stimulus modulated, show a degree of robustness in their coarse structure across stimulation conditions and during spontaneous activity [66]. Model neurons equipped with STDP robustly detect a pattern of currents encoded by the phase of a subset of afferents, even so when these patterns are presented at unpredictable intervals [76]. In this respect, one particular advantage of using oscillatory, rather than transient, activity patterns is that learning patterns referenced to the phase of oscillatory activity facilitates learning even when only a fraction of afferents are organized according to the phase [76].

Together, these observations corroborate the notion that biophysical mechanisms for transmitting, learning and decoding spike-timing information based on internal temporal frames codes exist and may be available within the microcircuitry of cortical sensory structures [72,77].
